# Audio-video recording during laparoscopic surgery reduces irrelevant conversation between surgeons: a cohort study

**DOI:** 10.1186/s12893-018-0428-x

**Published:** 2018-11-06

**Authors:** Hannah Bergström, Lars-Göran Larsson, Erik Stenberg

**Affiliations:** Department of Surgery, Faculty of Medicine and Health, Örebro University, SE-70185 Örebro, Sweden

## Abstract

**Background:**

The prevalence of perioperative surgical complications is a worldwide issue: In many cases, these events are preventable. Audio-video recording during laparoscopic surgery provides useful information for the purposes of education and event analyses, and may have an impact on the focus of the surgeons operating. The aim of the present study was to investigate how audio-video recording in the operating room during laparoscopic surgery affects the focus of the surgeon and his/her assistant.

**Methods:**

A group of laparoscopic procedures where video recording only was performed was compared to a group where both audio and video recordings were made. All laparoscopic procedures were performed at Lindesberg Hospital, Sweden, during the period August to September 2017. The primary outcome was conversation not relevant to the ongoing procedure. Secondary outcomes were intra- and postoperative adverse events or complications, operation time and number of times the assistant was corrected by the surgeon.

**Results:**

The study included 41 procedures, 20 in the video only group and 21 in the audio-video group. The material comprised laparoscopic cholecystectomies, totally extraperitoneal inguinal hernia repairs and bariatric surgical procedures. Irrelevant conversation time fell from 4.2% of surgical time to 1.4% when both audio and video recordings were made (*p* = 0.002). No differences in perioperative adverse event or complication rates were seen.

**Conclusion:**

Audio-video recording during laparoscopic abdominal surgery reduces irrelevant conversation time and may improve intraoperative safety and surgical outcome.

**Trial registration:**

Available at FOU Sweden (ID: 232771) and retrospectively at Clinical trials.gov (ID: NCT03425175; date of registration 7/2 2018).

## Background

Despite increased awareness of perioperative safety, the prevalence of intraoperative adverse events and postoperative complications remains an important issue, with perioperative complications being estimated to result in one million deaths each year. It is said that up to one half of all these are potentially preventable [1, 2]. Several steps could be taken to optimize the perioperative environment in order to reduce the effects of surgical trauma and to minimize unnecessary risks [3]. The use of a minimally invasive approach for standard surgical procedures is considered safe and has a low complication rate [4–6]. Laparoscopic surgery performed at a high volume center is associated with a lower complication rate than at a low-volume hospital [6, 7]. Even in ideal circumstances however, both intra- and postoperative adverse events do occur, causing significant morbidity and even mortality.

Routine video recording of surgical procedures has been reported to have a positive impact on the behavior of personnel and may improve surgical outcome [8–10]. Furthermore, recordings may be used for educational purposes [10] and can provide helpful information when serious complications occur [11]. It is feasible that simultaneous audio-video recording could further improve the educational value of these recordings, and may affect the behavior of surgeons performing the procedure. Since a higher intraoperative adverse event rate leads to a higher postoperative complication rate [6, 12], a reduction in irrelevant conversation thereby increasing focus during surgery may have a positive impact on the perioperative safety of patients.

The aim of this study was to evaluate whether the introduction of simultaneous audio and video recording during laparoscopic surgery has an impact on the focus of the surgeon and his/her assistant. We also considered the effect on complication and adverse event rates of these procedures.

## Methods

### Study sample

From August 29 until September 28, 2017, all laparoscopic surgical procedures performed at Lindesberg Hospital, Sweden, were consecutively included in the study. Due to the lack of previous data to base a Power calculation on, the study was planned as a pilot study aiming to include 20 operations in each of two groups – one with and one without audio recording. During the first three weeks of the study period, only standard video recording of the laparoscopic procedure was performed. Only the operation as seen from the laparoscope was recorded. During the second 3-week period, the surgeon and the assistant surgeon each had a microphone and both video (of the operation) and audio recordings were made during the procedure. This resulted in one period with video but no audio recording (control group) and one group with both video and audio recording (intervention group). No surgical procedure was excluded.

### Data collection

Data collection and recordings were performed by a single external observer. The observer was present in the operation room but not interfering with the operation team. The operation team consisted of two surgeons (one operating surgeon, and one assistant), two operation room nurses (one sterile and one non-sterile assistant), and two anesthesia nurses. A stopwatch was used to note the time when the surgeon and assistant surgeon were engaged in conversation considered irrelevant to the procedure. Conversations considered to be of educational purpose were not regarded as irrelevant. Registration of operation time and potential disturbances began following time-out, in accordance with the surgical safety checklist provided by the World Health Organization [13] (if this was used), and ended when the last stich was in place. Other items registered were: type of surgery; external disturbances; intraoperative adverse events; and whether or not the time-out before incision and sign-out after surgery checklist completion had been applied. Patient characteristics (age, gender, weight, length, body mass index, smoking habit), comorbidity (systemic disease, lung disease, diabetes, psychiatric disease, cardiovascular disease, pulmonary embolism/deep vein thrombosis), and data concerning postoperative complications were retrieved from the hospital clinical database.

### Outcome

The primary outcome was irrelevant conversation in seconds during the surgical procedure. Secondary outcomes were intra- and postoperative adverse events and complications, operation time and number of times the surgeon corrected the assistant. The Clavien-Dindo scale was used for grading postoperative complications [14]. Any deviation from the normal procedure was considered an intraoperative adverse event.

### Statistical analysis

Statistical analyses were performed using IBM SPSS Statistics for Macintosh, version 23. The Mann-Whitney *U* test was used to analyze continuous variables. The Chi-square or Fischer’s exact test were used for categorical variables depending on group size. *P*-values ≤0.05 were considered statistically significant.

### Ethics

This study was approved by the Regional Ethics Committee in Uppsala (Reference number 2017/247), and was conducted in accordance with the standards of the 1964 Helsinki Declaration and its later amendments [15].

## Results

During the inclusion period, 41 elective surgical procedures were observed and included in the study, no procedure was excluded. The surgical procedures performed in the control group (video recording only) were: laparoscopic gastric bypass and laparoscopic sleeve gastrectomy for morbid obesity; elective cholecystectomy; and totally extraperitoneal inguinal hernia repair. In the intervention group (audio-video recording) the surgical procedures performed were: laparoscopic gastric bypass; laparoscopic gastric sleeve; and elective cholecystectomy (Table 1). Median operation time was 63 min (IQR 54–97) in the control group and 73 min (IQR 63–76 min) in the intervention group.

**Table 1:** Surgical procedures in the control group and the intervention group

Surgical procedure	Control group	Intervention group
Laparoscopic gastric bypass	6 (30%)	10 (48%)
Laparoscopic gastric sleeve	2 (10%)	4 (19%)
Laparoscopic cholecystectomy	10 (50%)	7 (33%)
Total extraperitoneal inguinal hernia repair	2 (10%)	0

In the intervention group, there was a tendency towards higher body mass index, more patients had had previous abdominal surgery and less smoked (Table 2).

**Table 2:** Base-line characteristics

	Control group No audio recording *n* = 20	Intervention group Audio recording *n* = 21
Age, years^a^	50 (48–70)	*46 (36–51)*
Sex		
Female, *n* (%)	13 (65%)	14 (67%)
Male, *n* (%)	7 (35%)	7 (33%)
Active smoking, *n* (%)	5 (25%)	1 (5%)
Body Mass Index, kg/m^2,a^	30 (27–40)	*37 (31–38)*
Co-morbidity, *n* (%)	9 (45%)	10 (48%)
Hypertension, *n* (%)	3 (15%)	4 (19%)
Diabetes, *n* (%)	1 (5%)	4 (19%)
Pulmonary disease, *n* (%)	1 (5%)	4 (19%)
Cardiovascular disease, *n* (%)	3 (15%)	0 (0%)
Psychiatric disorder, *n* (%)	3 (15%)	2 (10%)
Systemic disease, *n* (%)	0 (0%)	3 (14%)
Previous abdominal surgery, *n* (%)	6 (30%)	11 (52%)

^a^Median (Interquartile range)

**Fig. 1:**
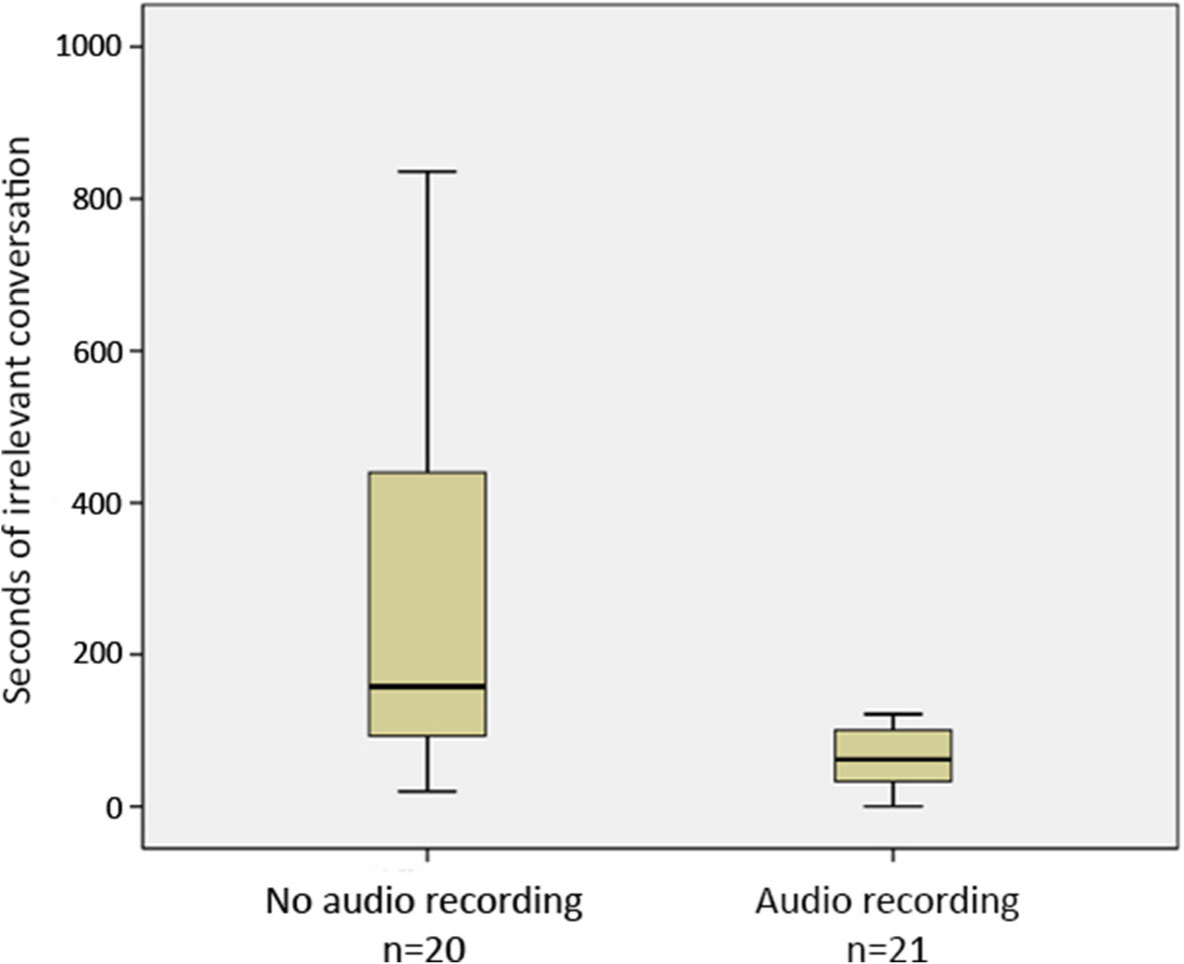
Time with irrelevant discussion during surgical procedures. Box-plot presenting time (seconds) with irrelevant discussion with or without audio-video recording

The time spent in irrelevant conversation was shorter in the intervention group (intervention group 62 s (IQR 29–100 s) compared to the control group 160 s (IQR 91–490 s), *p* = 0.002). With adjustment for operation time, the median percentage time spent in irrelevant conversation during surgery dropped from 4.2% (IQR 2.1–9.9%) to 1.4% (IQR 0.74–2.4%) when audio-video recordings were made during surgery (*p =* 0.002). Unadjusted time in irrelevant conversion is visualized in Fig. 1.

Only one patient suffered an intraoperative complication (ischemia of a small part of the small bowel secondary to planned division of the small bowel mesentery during a laparoscopic gastric bypass procedure). An intraoperative adverse event occurred during six procedures in the control group (30%), and during eight procedures (38%) in the intervention group (*p =* 0.59). Details of these events are listed in Table 3. No event was severe enough to result in postoperative morbidity. Blood loss during surgery was small (< 100 mL) and there was no need for blood transfusion.

**Table 3:** Intraoperative adverse event

	Control group No audio recording *n* = 20	Intervention group Audio recording *n* = 21
Perforation of gallbladder	4 (20%)	5 (24%)
Bleeding^a^	1 (5%)	2 (10%)
Serosal injury of small bowel	1 (5%)	0
Ischemia^b^	0	1 (5%)

^a^No bleeding resulted in > 100 ml blood loss

^b^The only event considered to be an intraoperative complication

One patient in the intervention group suffered from a postoperative complication. This patient was reoperated for undue postoperative pain, but no cause could be found and no intervention was made. The pain, however, improved after reoperation, and the event was thus considered a serious complication (Grade IIIb according to the Clavien-Dindo classification). No postoperative complication occurred in the control group.

The median number of corrections of the assistant was two (IQR 0.3–2.0) in the control group and one (IQR 1.0–2.0) in the intervention group (*p =* 0.53). The median number of external interferences during the procedure was 10 (IQR 4.3–15) in the control group and 11 (IQR 6.5–16) in the intervention group (*p =* 0.30).

## Discussion

Audio recording reduced irrelevant conversation time during surgery. Similar results have been seen in association with endoscopy [9], but to our knowledge this has not previously been investigated during laparoscopic surgery. The reduction in irrelevant conversation could indicate that the surgeon and his/her assistant were more focused on surgery. Although our study was too small to evaluate surgical outcome, reduction in irrelevant conversation could have had an impact on intraoperative adverse event rate, quality of surgery and ultimately, postoperative complication rate and long-term outcome. Indeed, a reduction in irrelevant conversation during closure of the abdominal wall has been shown to be associated with reduced risk for wound infection [16]. Although a link between irrelevant conversation and the occurrence of intraoperative adverse events remains to be studied, intraoperative adverse events are associated with an increased risk for postoperative complications [6, 12] and a reduction in the efficacy of surgical procedures [17].

Apart from reducing irrelevant conversation during surgical procedures, routine audio-video recording may be positive in other ways such as the creation of a database which may be used in the analysis of intraoperative events or near events; information that surgeons can learn from [18]. Recordings may also be used to enhance feedback in surgical training [18–20]. Furthermore, creating a surgical equivalent to the “black-box” of aviation may improve our understanding of the development of specific surgical complications and how these should be managed. An example is the occurrence of advanced common bile duct injury during laparoscopic cholecystectomy where audio-video recording of procedures provides much more information than surgical notes alone [21].

The major obstacles to routine audio recording in the operating room are mainly practical, ethical and perhaps legal. Audio-video recording in the working environment may affect the quality perceived, as well as the privacy of all personnel present and that of the patient. In addition, knowing that you are being recorded may add stress to the surgeon, in particular during complex maneuvers. The use of the recordings at a later stage must therefore be judicious. When used in surgical training and event analysis, it is possible for the material to be used to track errors of an individual with subsequent criticism or reprimand [22]. Furthermore, maintaining an audio-video recording database may be considered a threat to patient confidentiality. Is it really in the interest of the patient that recordings of everything happening in the operating room are made? There is also the concern that audio-video recordings can be used by the patient in legal claims against the hospital and the healthcare workers involved. Increased transparency, however, is generally beneficial to the healthcare provider [23], and is also in the interest of the patient. In fact as many as 81% of patients would like to have their procedure recorded and as many as 63% are willing to pay extra for this service [24].

Given the low intraoperative and postoperative complication rates in modern minimally invasive bariatric surgery [6, 25], laparoscopic cholecystectomy [5, 26] and TEP [27], the present study is by far too small to evaluate any impact of audio-video recording on perioperative complication rates. A different study design would be needed to address this end-point, and this was never the primary aim of the present study. The consecutive inclusion of procedures in this study resulted in slightly different distributions of surgical procedures within the two study groups. This resulted in differences in patient characteristics and operation times, but it is unlikely that this could have influenced irrelevant conversion during these procedures. All operations were audited by a single external observer present in the operating room. Manual auditing is known to affect the behavior of those who are being observed [28] and it is possible that this affected the time spent in irrelevant conversation. This effect, however, should have been the same in both groups. Surgeons and patients were blinded to the end-points of the study, but the observer was for practical reasons not blinded. The non-blinding of the observer adds a risk for observer bias. We have tried to reduce this risk by using an external observer not employed at the department at the time of the study, and without preceding opinions on pros and cons with audio-video recordings. Despite these efforts, this must still be considered a limitation with the study.

There are several potential benefits to be gained from audio-video recording during laparoscopic surgery, and the use of microphones is reported to be safe [29]. Surgeons should thus consider using routine audio-video recording during laparoscopic surgical procedures since this may improve intraoperative safety and surgical outcome.

## Conclusion

Audio-video recording during laparoscopic abdominal surgery reduces irrelevant conversation time and may improve intraoperative safety and surgical outcome.
